# Expanding the Host Range of Hepatitis C Virus through Viral Adaptation

**DOI:** 10.1128/mBio.01915-16

**Published:** 2016-11-08

**Authors:** Markus von Schaewen, Marcus Dorner, Kathrin Hueging, Lander Foquet, Sherif Gerges, Gabriela Hrebikova, Brigitte Heller, Julia Bitzegeio, Juliane Doerrbecker, Joshua A. Horwitz, Gisa Gerold, Sebastian Suerbaum, Charles M. Rice, Philip Meuleman, Thomas Pietschmann, Alexander Ploss

**Affiliations:** a Department of Molecular Biology, Princeton University, Princeton, New Jersey, USA; b Center for the Study of Hepatitis C, Laboratory of Virology and Infectious Diseases, The Rockefeller University, New York, New York, USA; c Institute for Experimental Virology, TWINCORE, Centre for Experimental and Clinical Infection Research, Hannover, Germany; d Center for Vaccinology, Ghent University, Ghent, Belgium; e Hannover Medical School, Hannover, Germany

## Abstract

Hepatitis C virus (HCV) species tropism is incompletely understood. We have previously shown that at the level of entry, human CD81 and occludin (OCLN) comprise the minimal set of human factors needed for viral uptake into murine cells. As an alternative approach to genetic humanization, species barriers can be overcome by adapting HCV to use the murine orthologues of these entry factors. We previously generated a murine tropic HCV (mtHCV or Jc1/mCD81) strain harboring three mutations within the viral envelope proteins that allowed productive entry into mouse cell lines. In this study, we aimed to characterize the ability of mtHCV to enter and infect mouse hepatocytes *in vivo* and *in vitro*. Using a highly sensitive, Cre-activatable reporter, we demonstrate that mtHCV can enter mouse hepatocytes *in vivo* in the absence of any human cofactors. Viral entry still relied on expression of mouse CD81 and SCARB1 and was more efficient when mouse CD81 and OCLN were overexpressed. HCV entry could be significantly reduced in the presence of anti-HCV E2 specific antibodies, suggesting that uptake of mtHCV is dependent on viral glycoproteins. Despite mtHCV’s ability to enter murine hepatocytes *in vivo*, we did not observe persistent infection, even in animals with severely blunted type I and III interferon signaling and impaired adaptive immune responses. Altogether, these results establish proof of concept that the barriers limiting HCV species tropism can be overcome by viral adaptation. However, additional viral adaptations will likely be needed to increase the robustness of a murine model system for hepatitis C.

## IMPORTANCE

At least 150 million individuals are chronically infected with HCV and are at risk of developing serious liver disease. Despite the advent of effective antiviral therapy, the frequency of chronic carriers has only marginally decreased. A major roadblock in developing a vaccine that would prevent transmission is the scarcity of animal models that are susceptible to HCV infection. It is poorly understood why HCV infects only humans and chimpanzees. To develop an animal model for hepatitis C, previous efforts focused on modifying the host environment of mice, for example, to render them more susceptible to HCV infection. Here, we attempted a complementary approach in which a laboratory-derived HCV variant was tested for its ability to infect mice. We demonstrate that this engineered HCV strain can enter mouse liver cells but does not replicate efficiently. Thus, additional adaptations are likely needed to construct a robust animal model for HCV.

## INTRODUCTION

At least 150 million individuals worldwide are chronically infected with hepatitis C virus (HCV). Chronic HCV infection is associated with severe liver disease, including fibrosis, cirrhosis, and hepatocellular carcinoma. Treatment options have drastically improved in recent years, and it is now possible to effectively cure HCV infection in the majority of patients. However, effective ways to prevent infection, for example via vaccination, remain elusive. To systematically test and prioritize vaccine candidates, development of an immunocompetent animal model, optimally with inheritable susceptibility to HCV, remains a high priority.

HCV exhibits a narrow and mechanistically incompletely understood species tropism. While humans and chimpanzees are readily susceptible to HCV infection, most other species—with the exception of intermittent, sporadic viremia in tree shrews—appear to be resistant (reviewed in reference 1). Resistance of mice to HCV is multifactorial and determined at least by blocks in viral entry and replication (reviewed in reference 2). HCV utilizes a large number of host factors to enter its host cells, the human hepatocyte (reviewed in reference 3). These factors include glycosaminoglycans (GAGs) present on heparan sulfate proteoglycans (HSPGs), low-density-lipoprotein receptor (LDLR) (4, 5), CD81 (6), scavenger receptor class B member 1 (SCARB1) (7), the tight junction claudin proteins, including claudin-1 (CLDN1) (8) and CLDN6 and CLDN9 (9), occludin (OCLN) (10, 11), the receptor tyrosine kinases epidermal growth factor receptor (EGFR), and ephrin receptor A2 (EphA2) (12), the cholesterol transporter Niemann-Pick C1-like 1 (NPC1L1) (13), transferrin receptor 1 (TfR1) (14), and the cell death-inducing DFFA-like effector b (CIDEB) (15). It was previously shown that CD81 (6), SCARB1 (7) and the two tight junction proteins CLDN1 (8) and OCLN (10, 11) are all required for uptake into human and also rodent cells but that only CD81 and OCLN need to be of human origin to facilitate this process (11). Indeed, transient adenoviral (16) or stable transgenic (17) expression of human CD81 and OCLN in mouse hepatocytes facilitates HCV uptake. The differential abilities of mouse and human CD81 and OCLN to support viral entry have been mapped to amino acids in their second extracellular loops (11, 18). While infectious HCV particles can assemble in mouse liver cells *in vitro* (19) and *in vivo* (17), HCV RNA replication in mice is limited, presumably by a combination of innate antiviral immune responses (17, 20–23) and possibly by poor compatibility between murine orthologues of replication cofactors and the virally encoded components of the HCV replication machinery (1).

Complementary to the host genetic adaptation approach, adaptation of HCV to rodent hosts is an alternative strategy for establishing a mouse model for hepatitis C. We previously used an unbiased selection approach to adapt an HCV genotype 2a strain, Jc1, to use mouse CD81 (24). We identified three adaptive mutations in the HCV envelope proteins E1 and E2 that facilitated uptake into cell lines expressing human SCARB1, CLDN1, OCLN, and mouse, rat, or hamster CD81 (24). The mutations significantly increased the affinity of the virus for the large extracellular loops of human CD81, suggesting an indirect enhancement by exposing a CD81 binding site. The mutations in the mouse CD81 (mCD81)-adapted, i.e., murine tropic, virus (mtHCV or Jc1/mCD81) altered usage of human SCARB1 (hSCARB1) and human OCLN (hOCLN). Blocking antibodies against hSCARB1 and silencing of hOCLN had a less pronounced effect on the entry of the mutant virus compared to the parental strain, suggesting that the mCD81-adapted virus was less dependent on hSCARB1 and hOCLN. Finally, mouse fibroblasts expressing murine CD81, SCARB1, CLDN1, and OCLN supported the uptake of adapted virus. This entry could be blocked with anti-mCD81 antibodies, indicating that the species-specific restriction to human OCLN was altered while dependence on CD81 was maintained.

Here, we aimed to extend this work and directly test whether our mtHCV harboring mutations that facilitate efficient engagement of murine CD81 and OCLN could infect murine primary hepatocytes *in vitro* and *in vivo*. We demonstrate that mtHCV can enter mouse hepatocytes in cell culture and that this uptake is dependent on the adaptive mutations present in HCV E1 and E2. Using a sensitive reporter, we show that mtHCV enters mouse hepatocytes *in vivo* in an HCV glycoprotein-dependent manner. Although hepatic overexpression of mouse CD81 and OCLN enhances HCV entry, uptake is not dependent on ectopic expression, and mtHCV is capable of utilizing the endogenous proteins, albeit at low efficiency. Previous data suggest that innate immunity restricts HCV replication in mouse hepatocytes *in vitro* (20–22, 25) and *in vivo* (17). Mice with targeted disruptions of signal transducer and activator of transcription factor 1 (STAT1), which have severely impaired type I and III interferon (IFN) responses, did not develop persistent viremia following infection. Engraftment of STAT1-deficient mouse hepatocytes into immunodeficient mice with liver injuries allowed us to directly test whether murine adaptive immune responses further antagonize HCV replication. However, in the absence of functional B, T, and natural killer cells, mtHCV infection in murine hepatocytes was not further enhanced. Collectively, these data suggest that additional barriers limit propagation of mtHCV in mice. The low uptake efficiency of mtHCV into mouse hepatocytes expressing endogenous levels of viral entry factors may preclude efficient spread, which may be necessary to establish persistence. In addition, the efficiency of postentry steps of the viral life cycle could conceivably be improved with human host factors important for HCV replication, assembly, and/or egress. Thus, additional, currently unknown proviral factors and/or negative regulators antagonizing HCV will have to be identified to enhance HCV replication and assembly in mouse cells.

## RESULTS

### mtHCV-specific adaptations are maintained upon long-term replication *in vivo.*

It was previously demonstrated that cell culture adaptive mutations that increased the level of viral replication *in vitro* could potentially compromise viral fitness *in vivo* (26). Thus, we aimed to test whether the three adaptive mutations in Jc1/mCD81 affected its viral fitness *in vivo* and whether they were stably maintained during long-term *in vivo* replication.

**FIG 1:**
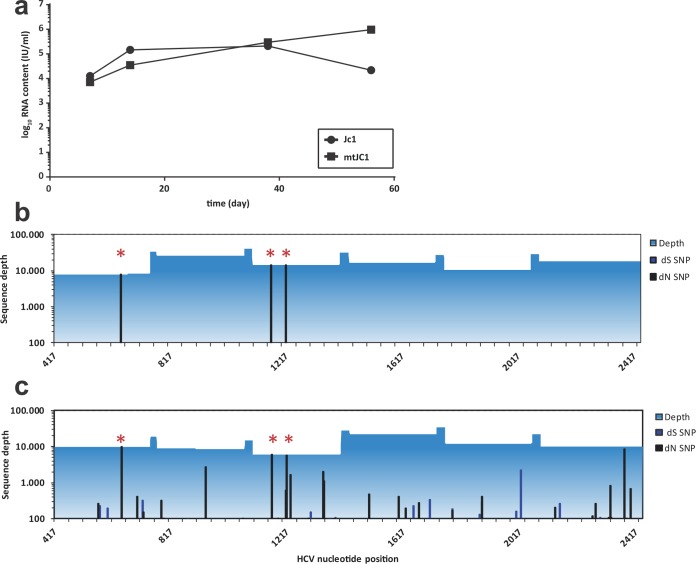
Evolution of mtHCV during long-term replication in uPA-SCID mice repopulated with human hepatocytes. (a) Mice were inoculated with an equal dose of wild-type HCV and mtHCV. Virus plasma levels were determined at the indicated time points postinoculation. (b and c) Evolution of mtHCV sequence during chronic infection in mice. The E1-E2-p7 coding region of the virus inoculum (b) and of the virus population circulating 8 weeks postinoculation (c) was amplified and sequenced using the Roche 454 platform. The *x* axis represents nucleotide position relative to the reference sequence. The *y* axis represents nucleotide coverage across the E1-E2-p7 coding region with synonymous (dS) and nonsynonymous (dN) single nucleotide polymorphism (SNP) frequencies in the viral population relative to the reference sequence highlighted. The mtHCV-specific adaptations L216F, V388G, and M405T are marked with red asterisks.

We took advantage of a previously established human liver-uPA-SCID mouse model that stably supports HCV replication (27, 28). Stable engraftment with human hepatocytes was determined by monitoring serum levels of human albumin (data not shown). We infected the mice with 6 × 10^5^ TCID_50_ (50% tissue culture infective doses) of Jc1 or Jc1/mCD81 and observed stable and comparable HCV RNA levels for both Jc1- and Jc1/mCD81-infected mice for about 56 days (Fig. 1a). Eight weeks after inoculation of Jc1/mCD81, we deep sequenced the genes of the envelope glycoproteins E1 and E2, the signal peptides of E1 and p7. All three adaptive mutations—L216F, V388G, and M405T—were conserved 100% between the viral inoculum (Fig. 1b) and the mouse-passaged virus (Fig. 1c). Taken together with the observation that infection with both Jc1 and Jc1/mCD81 leads to comparable HCV RNA serum levels, these data demonstrate that the three adaptive mutations do not diminish the replication capacity of Jc1/mCD81 *in vivo*. Furthermore, neither virus acquired any additional adaptive mutation(s) upon long-term *in vivo* replication.

### Jc1/mCD81 is capable of entering murine hepatocytes *in vitro* and *in vivo.*

**FIG 2:**
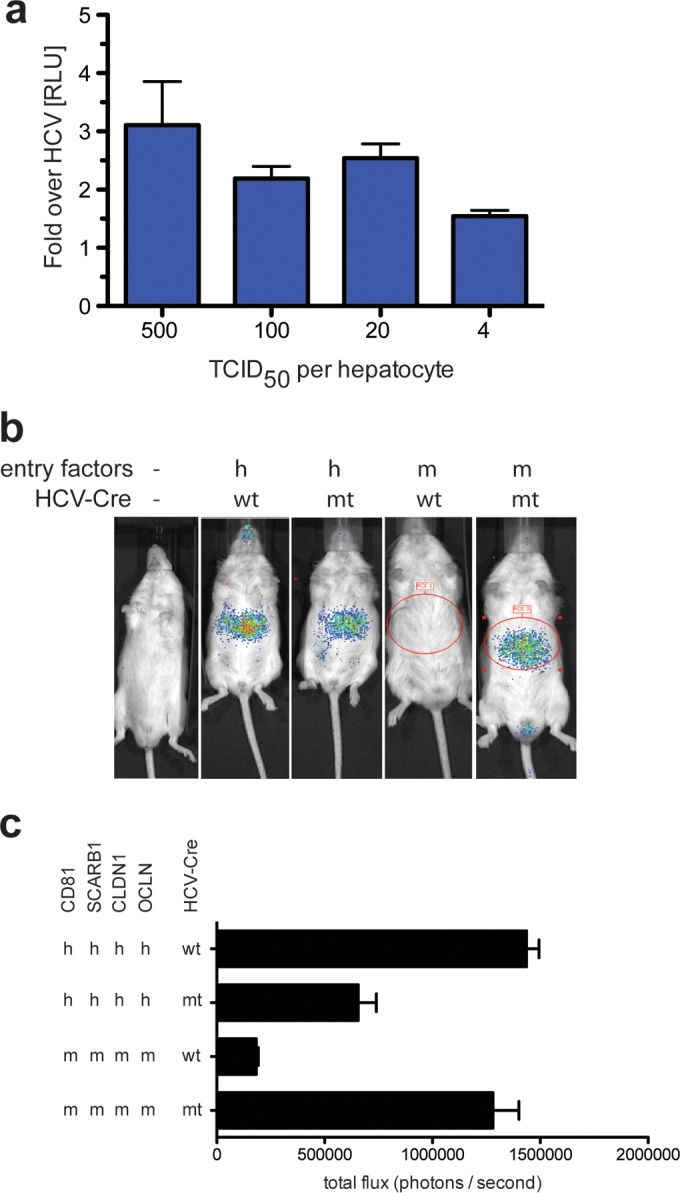
mtHCV can enter mouse hepatocytes *in vitro* and *in vivo*. R26-LSL-Fluc mice were injected with 10^11^ adenoviral particles encoding human (h) or mouse (m) CD81, SCARB1, CLDN, and OCLN. Bioluminescence was measured at 72 h postinjection of 2 × 10^7^ 50% tissue culture infectious doses (TCID_50_) of cell culture-produced murine tropic (mt) or wild-type (wt) HCV-Cre. (a) Primary hepatocytes isolated from C57BL/6 mice were plated on collagen-coated plates, and monolayers were infected with Jc1(p7nsGluc2A) or Jc1/mCD81Gluc2A with the indicated numbers of infectious particles (as titered on Huh-7.5 cells) per primary hepatocyte. Luciferase activity was measured 48 h postinfection. Data are represented as the signal of mtHCV over the parental strain. (b) Representative pseudo-colored images. (c) Quantitation of total photon flux/second (*n* ≥ 4). Values are means plus standard deviations (SD) (error bars).

We previously demonstrated that ectopic expression of the murine orthologues of the four canonical HCV entry factors—SCARB1, CD81, CLDN1, and OCLN—supports cell entry of HCV pseudoparticles (HCVpp) packaged with envelopes of mtHCV (mtHCVpp) (24). However, initial attempts to quantify mtHCVpp uptake into primary murine hepatocytes in cell culture failed (24). Since HCV RNA replication is inefficient in murine hepatocytes, we adopted a Jc1 genome expressing a *Gaussia* luciferase reporter [Jc1(p7nsGluc2A)] (29) that allows highly sensitive detection of HCV entry by luciferase secretion following initial translation of the HCV genome. We engineered the three adaptive mutations of mtHCV into Jc1(p7nsGluc2A), yielding Jc1/mCD81(p7nsGluc2A). Infection of mouse primary hepatocytes isolated from C57BL/6 mice with Jc1/mCD81(p7nsGluc2A) resulted in a 2- to 4-fold-higher luciferase signal than infection with the parental virus (Fig. 2a), suggesting that the three adaptive mutations enabled viral uptake, albeit at low efficiency.

To directly test whether mtHCV is capable of entering mouse hepatocytes *in vivo*, we utilized a highly sensitive reporter in which cellularly encoded firefly luciferase is activated by Cre recombinase expressed in a bicistronic HCV genome configuration (BiCre-Jc1) (16). Analogous to previous studies, we expressed human or mouse CD81, SCARB1, CLDN1, and OCLN via an adenoviral vector in the livers of Rosa26-Fluc mice (30) and infected animals with BiCre-Jc1 or BiCre-Jc1/mCD81. In accordance with previously published data, BiCre-Jc1 entered mouse hepatocytes overexpressing the human orthologues of the canonical entry factors but not mouse hepatocytes overexpressing murine orthologues of the canonical entry factors. In contrast, the bicistronic HCV genome harboring the adaptive mutations in E1 and E2 activated the reporter equally well in mice overexpressing entry factors from either humans or mice (Fig. 2b and c). Collectively, these data demonstrate that the three adaptive mutations in E1 and E2 enable uptake of HCV into mouse primary hepatocytes *in vitro* and *in vivo*.

### Efficiency of mtHCV uptake *in vivo* positively correlates with the viral dose and expression levels of HCV entry factors.

**FIG 3:**
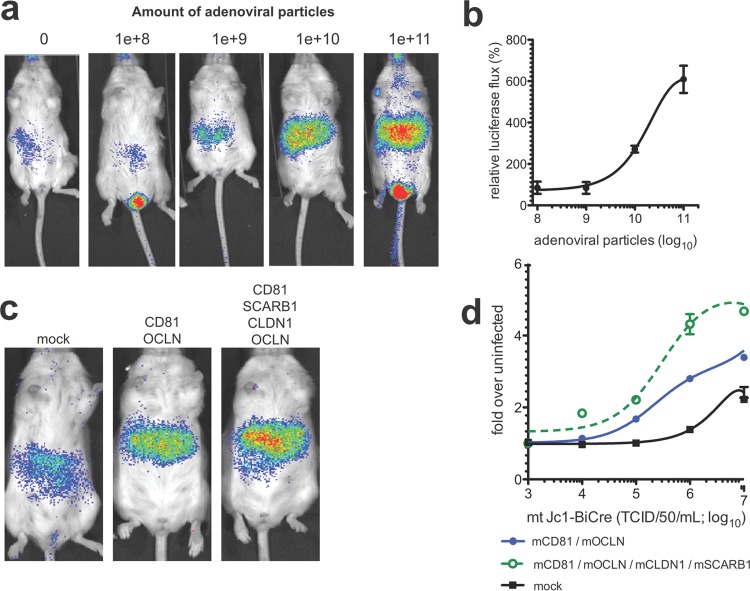
Efficiency of mtHCV uptake positively correlates with HCV entry factor expression levels and challenge dose. (a and b) Rosa26-Fluc mice were injected with limiting dilutions of adenoviruses encoding mouse CD81 (mCD81), SCARB1, CLDN1, and OCLN prior to infection with 1 × 10^6^ TCID_50_ mtHCV-Cre. (c and d) Rosa26-LSL-Fluc mice were injected with 10^11^ particles of adenoviruses encoding mouse CD81, SCARB1, CLDN1, and OCLN or mock injected, followed 24 h later by infection with the indicated doses of mtHCV-Cre or, as a control, PBS (*n* = 3). (a and c) Representative pseudocolored images; (b and d) quantitation of bioluminescent signal (*n* = 3). Data were acquired 72 h following HCV infection. Values are means ± SD (error bars).

To assess the efficiency of mtHCV uptake *in vivo*, we performed dose-response experiments titrating either the level of overexpression of mouse CD81, SCARB1, CLDN1, and OCLN while keeping the viral inoculum constant (Fig. 3a and b) or overexpressing mouse canonical entry factors and varying the inoculum dose (Fig. 3c and d). Rosa26-Fluc mice were injected intravenously with low (1 × 10^8^ adenoviral [AdV] particles/mouse), intermediate (1 × 10^9^ or 1 × 10^10^ AdV particles/mouse), or high (1 × 10^11^ AdV particles/mouse) doses of adenoviral particles or no adenovirus and subsequently infected with cell culture-produced BiCre-Jc1/mCD81 (2 × 10^7^ TCID_50_/mouse). The highest expression of HCV entry factors enhanced the signal of the luminescent reporter 6- to 7-fold compared with mice that received the lowest dose of HCV entry factors (Fig. 3a and b). These data are in accordance with our previous data demonstrating that uptake of HCV with native E1/E2 glycoproteins is more efficient in mouse hepatocytes expressing high levels of human CD81, SCARB1, CLDN1, and OCLN (16).

Of note, even in the absence of ectopic expression of HCV entry factors, mtHCV was still capable of entering mouse hepatocytes, albeit at very low levels (Fig. 3a, left panel). This suggests that endogenous expression levels of HCV entry factors in the mouse liver may suffice for viral uptake.

We observed a similar correlation when mice overexpressing mouse CD81, SCARB1, CLDN1, and OCLN or just CD81 and OCLN were infected with various doses of cell culture-produced BiCre-Jc1/mCD81. The higher the inoculum, the more robustly the luminescent reporter was activated in mice overexpressing murine versions of all four HCV entry factors (ca. 4- to 5-fold over background) (Fig. 3c and d). Because viral uptake relied on endogenous expression of SCARB1 and CLDN1, in mice overexpressing only mouse CD81 and OCLN, the signal was about 3-fold over background. In mice not injected with HCV entry factor-expressing adenoviruses, luminescent reporter activity was even lower, only 2-fold over background levels (Fig. 3d).

Collectively, these data demonstrate that mtHCV entry into murine hepatocytes is more efficient when mouse CD81, SCARB1, CLDN1, and OCLN are not limiting. It should be noted that these constraints are due in part to the detection limit of the Cre-based reporter assay.

### Quantification of the frequency of HCV-infected cells.

**FIG 4:**
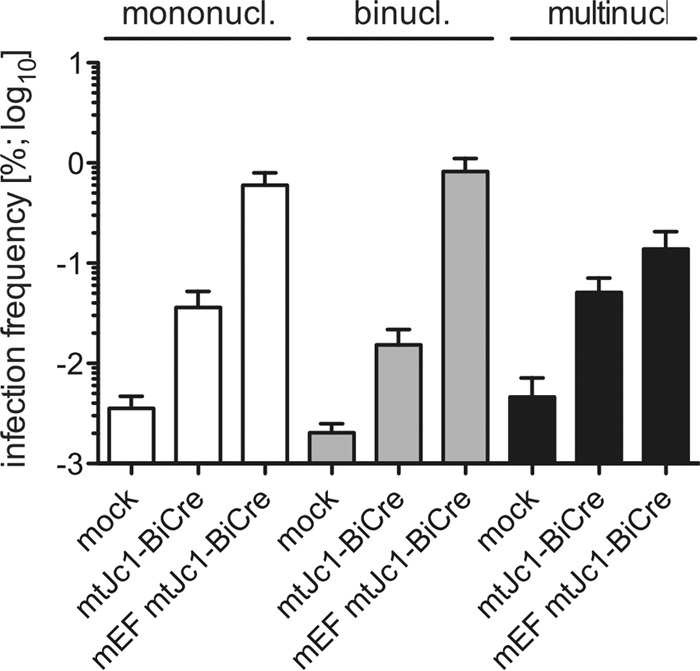
Quantitation of the frequency of mtHCV-infected hepatocytes *in vivo*. Rosa26-LSL-GNZ mice were injected with 1 × 10^11^ adenoviral particles encoding mouse entry factors (mEF) or not injected with AdV and subsequently infected with 1 × 10^6^ TCID_50_ mtHCV-Cre. Seventy-two hours following infection, single-cell suspensions were prepared by liver perfusion with collagenase. Cells were counterstained with DRAQ5 to distinguish between mono-, bi-, and multinucleated (or multiploid) cells. The total frequency of infected hepatocytes within the subpopulations was determined by flow cytometry (*n* = 3). Values are means plus SD.

To estimate the number of HCV-infected liver cells, we used an indicator mouse strain in which Cre leads to activation of a nucleus-localized green fluorescent protein (GFP)/β-galactosidase (GNZ) reporter (Rosa26-GNZ) (31). In approximately 0.07 to 0.09% of mouse hepatocytes, GFP fluorescence was detected when uptake was solely mediated by the endogenous murine entry factors after a challenge with a dose of 2 × 10^7^ TCID_50_ (Fig. 4). Upon adenoviral overexpression of mouse CD81, SCARB1, CLDN1, and OCLN, the frequency increased roughly 10-fold, reaching levels that were about one-half of those reported in mice expressing human orthologues of the canonical entry factors (1 to 1.5%) (16, 17). Following previously established protocols (32), we also included DNA intercalating dyes (Hoechst) to determine any differences in the ability of mono-, bi-, and multinucleated hepatocytes to support HCV uptake. Infection frequencies were largely equivalent among these subsets of hepatocyte populations, with slightly reduced numbers in multi- versus mono- or binucleated cells.

### mtHCV enters hepatocytes *in vivo* in a glycoprotein-dependent manner.

**FIG 5:**
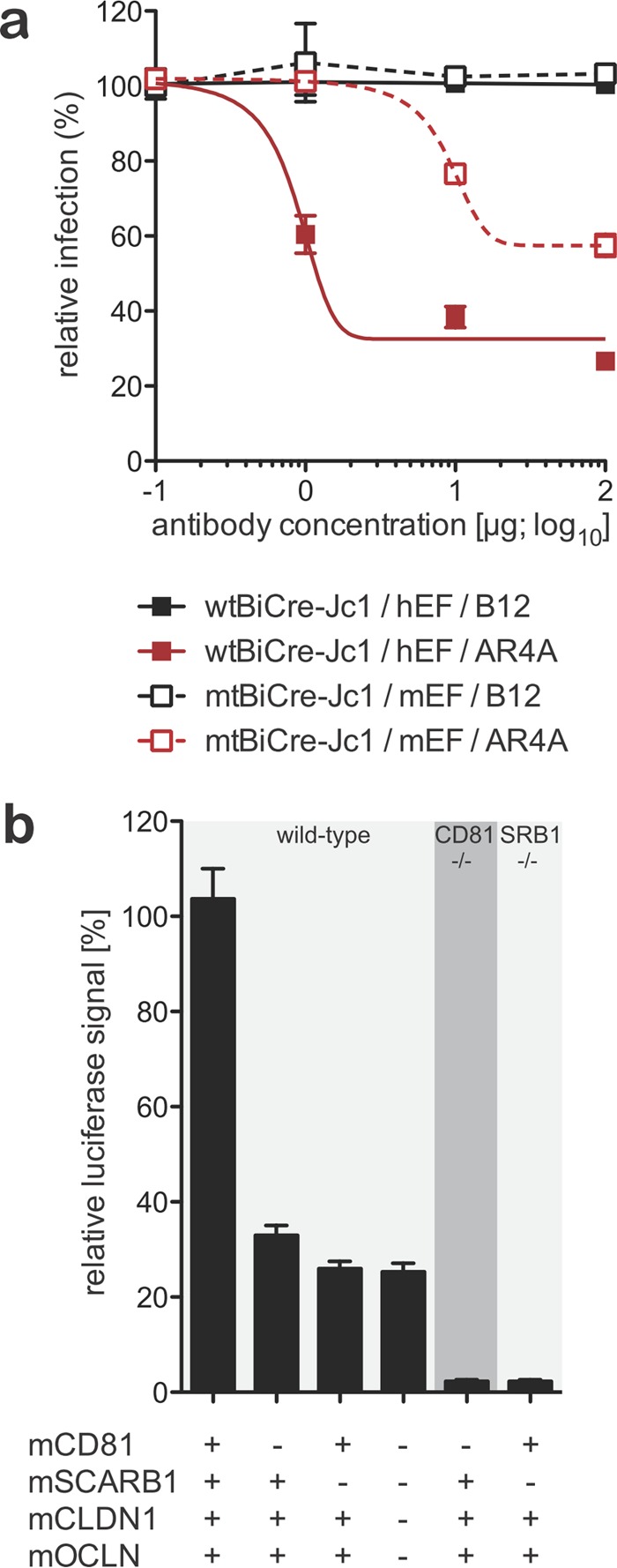
Uptake of mtHCV *in vivo* can be blocked with anti-HCV E2-specific antibodies and is dependent on endogenous expression of mouse CD81 and SCARB1. (a) R26-LSL-Fluc mice were injected with 1 × 10^11^ particles of adenoviruses expressing mouse CD81, SCARB1, CLDN1, and OCLN. mtHCV-Cre was incubated with the indicated doses of anti-HCV E2 (clone AR4A) or an anti-HIV isotype control antibody (clone B12) for 1 h prior to injection (1 × 10^6^ TCID_50_) and 24 h after adenoviral delivery (*n* = 3). Data were acquired 72 h following HCV infection. Values are means ± SD. (b) Rosa26-Fluc mice were crossed with mCD81^−/−^ or mSCARB1^−/−^ mice, and offspring with the indicated zygosities for the respective mutant alleles were injected with the indicated combinations of AdVs encoding mouse CD81 (mCD81), SCARB1, CLDN1, and OCLN and 24 h later with 2 × 10^7^ TCID_50_ BiCre-Jc1 (*n* ≥ 4). Luminescence was quantified 72 h following HCV infection. Values are means plus SD.

To demonstrate that mtHCV entry into mouse hepatocytes is dependent on the interaction of the viral envelope with cellularly encoded receptors, we performed blocking and loss-of-function experiments (Fig. 5). First, we took advantage of the well-characterized broadly neutralizing antibody AR4A, which binds to an epitope in the HCV envelope protein E2 (33). mtHCV was preincubated with various concentrations of either AR4A or an isotype control antibody (B12) and injected into Rosa26-Fluc mice expressing mouse CD81, SCARB1, CLDN1, and OCLN. Blockade with AR4A led to a 30 to 40% decrease in the luciferase signal at the highest antibody concentrations (Fig. 5a). For comparison, preincubation of the parental BiCre-Jc1 strain resulted in comparable, although slightly more effective (60 to 70%), blockade in Rosa26-Fluc mice expressing human CD81, SCARB1, CLDN1, and OCLN (Fig. 5a). These data suggest that uptake of HCV and mtHCV engaging human or mouse HCV entry factors, respectively, is dependent on HCV glycoproteins.

To further explore this process, we employed mice with targeted disruptions in the genes encoding CD81 (CD81^−/−^) or SCARB1 (SCARB1^−/−^) that we crossed to the Rosa26-Fluc background (Fig. 5b). We focused our analysis on these two molecules since there is strong evidence that both CD81 and SCARB1 interact directly with lipoviral HCV particles, unlike many of the other HCV entry factors. CD81^−/−^ and SCARB1^−/−^ mice and, as controls, mice with both alleles were injected with adenoviruses delivering the indicated combinations of the canonical entry factor mouse orthologues. In CD81^−/−^ mice overexpressing mouse SCARB1, CLDN1, and OCLN (Fig. 5b, 5th column from the left), the uptake efficiency was reduced by 90% compared with mice with the endogenous CD81 and treated with the same combination of adenoviruses (Fig. 5b, 2nd column from the left). Likewise, in SCARB1^−/−^ mice overexpressing mouse CD81, CLDN1, and OCLN (Fig. 5b, 6th column from the left), the uptake efficiency was reduced by ca. 85% compared to SCARB1^+/+^ mice treated with the same combination of adenoviruses (Fig. 5b, 3rd column from the left). While previous *in vitro* experiments using anti-SCARB1 blocking suggested that mtHCV entry may be less dependent on SCARB1, our *in vivo* experiment establishes that mtHCV requires both CD81 and SCARB1 to enter murine hepatocytes *in vivo*.

### mtHCV does not replicate in STAT1-deficient mice.

**FIG 6:**
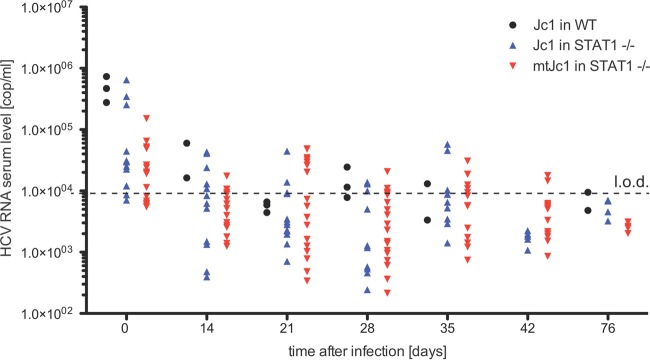
Infection of mice with blunted innate immunity does not result in persistent infection. Mice with a fully intact innate immune system (wild-type [WT]) or lacking STAT1 were infected with 5 × 10^6^ TCID_50_ of Jc1 or Jc1/mCD81. HCV RNA levels (number of copies [cop] per milliliter) were measured by qRT-PCR at the indicated time points. Each symbol represents the value f or an individual animal. The dashed line shows the limit of detection (l.o.d.).

To test whether mtHCV may not only enter but also go through later stages of the viral life cycle, we analyzed postentry steps *in vivo*. Previous studies demonstrated that antiviral defenses, in particular type I and III IFN-dependent pathways, interfere with HCV RNA replication in mouse cell lines and in mice (17, 20–23, 33, 34). Thus, we tested the replicative fitness of mtHCV in mice with a targeted disruption of STAT1 (Fig. 6). STAT1 knockout mice are severely impaired in type I and III IFN signaling. We previously showed that STAT-deficient mice transgenically expressing human CD81, SCARB1, CLDN1, and OCLN support low levels of HCV infection over several weeks (17). While we were also able to detect HCV RNA—presumably from the residual inoculum—4 h following infection of STAT1^−/−^ mice with cell culture-produced mtHCV (5 × 10^6^ TCID_50_/mouse, intravenously), none of the animals were consistently viremic at any of the later time points. Furthermore, we did not detect any differences between STAT1^−/−^ and isogenic wild-type control animals.

### Minimal evidence for mtHCV RNA replication in the absence of antiviral innate and adaptive immune responses.

It is conceivable that those cells in which mtHCV may start to replicate are rapidly cleared by the murine adaptive immune system. Thus, to test whether mtHCV may establish persistent infection in highly immunocompromised animals in which both innate and adaptive responses were compromised, we transplanted Alb-uPA SCID/beige mice (28) with hepatocytes isolated from STAT1^−/−^ mice. Consistent with previously published data (35), transplantation of 2 × 10^5^ or 4 × 10^5^ mouse hepatocytes from STAT1^−/−^ mice rescued Alb-uPA SCID/beige mice from acute liver failure and resulted in successive weight gain (data not shown). Stably engrafted animals were injected with cell culture-produced mtHCV (1.33 × 10^6^ TCID_50_), and viremia was monitored for 8 weeks. In contrast to human liver chimeric mice in which mtHCV caused high-level, persistent viremia (Fig. 1), most murine liver chimeric mice remained below the limit of detection (Table 1). A single mouse (B620R) became viremic at a single time point 4 weeks following inoculation, but viremia was just slightly above the limit of detection of 300 IU/ml. These data suggest that mtHCV does not replicate in murine hepatocytes or replicates at levels below the detection limit of our assays.

**TABLE 1:** Infection of uPA-SCID mice engrafted with STAT1-deficient murine hepatocytes with mtHCV does not result in stable viremia^a^

Group	Mouse ID^b^	Hepatocyte donor	Hepatocyte dose	Viral RNA (IU/ml) at the following time postinfection (wk)^c^:				
				2	3	4	5	8
RO	K1389L	STAT-1 KO	2.10E+5	<375	<300	<300	<300	<150
	K1389RL	STAT-1 KO	2.10E+5	<375	<300	<300	<300	<150

IP	B620	STAT-1 KO	4.10E+5	<375	<300	<300	<300	<150
	B620L	STAT-1 KO	4.10E+5	<375	<300	<300	<300	<150
	B620R	STAT-1 KO	4.10E+5	<375	<300	**+, <300**	<300	<150

Negative control (RO)	B623L	Wild-type		<375	ND	ND	ND	ND

a uPA-SCID mice were injected with 2 × 10^5^ or 4 × 10^5^ murine hepatocytes derived from STAT1-deficient (knockout [KO]) mice. Resultant cohorts (RO and IP) were infected intravenously with 1.3 × 10^6^ TCID_50_ mtHCV-Cre.

b ID, identifier.

c HCV RNA levels in serum were measured for up to 8 weeks postinfection. Viral RNA was quantified for up to 8 weeks postinfection using the Roche COBAS AmpliPrep instrument and the COBAS TaqMan48 HCV test. The limit of detection was 300 IU/ml. ND, not determined.

## DISCUSSION

The molecular basis for HCV’s narrow host range remains incompletely understood. This has posed challenges for developing small animal models for HCV infection. In rodent cells, the HCV life cycle is blocked at the level of entry and at postentry steps. HCV’s inability to enter rodent cells can be explained by differences in critical residues in the second extracellular loops of CD81 (36, 37) and OCLN (18). Consequently, expression of human CD81 and OCLN, along with human or mouse SCARB1 and CLDN1, facilitates HCV uptake in mouse cells *in vitro* (11) and *in vivo* (16, 17, 38).

As an alternative to genetic host adaptations, we have previously demonstrated that the block of HCV at the level of entry can also be overcome by viral adaptation (24). Using an *in vitro* selection approach, we identified mutations within HCV E1 and E2 that increased the affinity of the viral envelope for mouse CD81. These mutations appeared to more broadly affect the conformation of the viral envelope, as the resulting mCD81-adapted strain was also less dependent on human OCLN and could enter cell lines expressing only mouse CD81, SCARB1, CLDN1, and OCLN (24). Here, we show that Jc1/mCD81 can also infect murine primary hepatocytes *in vitro* and *in vivo*. Uptake of this mtHCV strain is dependent on interactions of the viral glycoproteins with murine orthologues of HCV entry factors, specifically CD81 and SCARB1. However, uptake of mtHCV into mouse hepatocytes appears to be rather inefficient, as viral uptake can be detected in only 0.05 to 0.1% of murine hepatocytes (Fig. 4). This is further corroborated by our observation that adenoviral overexpression of the murine orthologues increased uptake efficiency (Fig. 3). It is conceivable that additional adaptive mutations may be necessary to facilitate more efficient entry. At the same time, it cannot be ruled out that molecules present on the surfaces of mouse hepatocytes but presumably not human hepatocytes interfere with viral uptake.

We further explored whether Jc1/mCD81 could also proceed through other steps of the viral life cycle. Numerous *in vitro* studies have demonstrated that type I and III IFN-dependent antiviral defenses appear to act more efficiently to shut down HCV infection in rodent cells than in human cells (25, 39). For example, fibroblast and immortalized hepatic cell lines from mice deficient in interferon regulatory factor 3 (IRF3), IRF7, mitochondrial antiviral signaling protein (MAVS), and STAT1 support HCV RNA replication more efficiently than wild-type cells do (17, 20–23). Thus, we employed STAT1-deficient mice, which are severely impaired in type I and III IFN signaling and animals lacking both functional innate and adaptive immune responses. We previously showed that STAT^−/−^ mice transgenically expressing human CD81, SCARB1, CLDN1, and OCLN became viremic, albeit at low levels (17). Other studies in HCV entry factor transgenic mice on an ICR background suggested that HCV infection could become chronic even in fully immunocompetent animals (38).

Blunting cell intrinsic immunity and even both innate and adaptive immunity was not sufficient to establish persistent HCV infection. This may be explained by a combination of the very low entry efficiency and replication of mtHCV in mouse hepatocytes in the absence of ectopic (over)expression of the human orthologues of HCV entry factors. Future efforts will have to focus on identifying putatively missing human-specific positive regulators of HCV replication in mouse hepatocytes or alternatively, identifying mutations that increase HCV replicative fitness in mouse cells. The latter approach has been attempted, but mutations that arose during replication of antibiotic-resistant HCV replicons in mouse cells seemed to be random rather than adaptive (40), as none substantially enhanced replication efficiency in the murine cell environment when reintroduced into the parental subgenome (41).

Conceivably, it may be simpler to adapt HCV to other species that are genetically more closely related to humans than mice. Results from prior studies suggested that several other nonhuman primate species are resistant to HCV infection (42, 43). However, more-recent work has shown that hepatocyte-like cells derived from pig-tailed macaques induced pluripotent stem cells (44) and primary adult hepatocytes from rhesus macaques (45) can support the entire HCV life cycle. When rhesus macaque hepatocytes were engrafted in immunodeficient liver injury recipients, the resultant simian liver chimeric mice supported persistent HCV infection, albeit with delayed kinetics and lower peak viremia levels than in control humanized mice. These data suggest that it may be possible to establish HCV infection in species more distantly related to humans. However, since mice and humans diverged phylogenetically approximately 65 million years ago, it may be rather challenging to bridge the genetic gap by viral adaptation despite HCV’s considerable genetic plasticity.

Over the last few years, other viruses related to HCV in either the *Pegivirus* genus, also a part of the *Flaviviridae* family, or the *Hepacivirus* genus have been identified in a variety of species, including dogs (46), horses (47, 48), wild mice (49), and rats (50). An in-depth understanding of how these viruses establish chronicity in species not known to be zoonotic reservoirs for HCV may guide further, more directed adaptations of HCV to robustly infect heterologous hosts.

## MATERIALS AND METHODS

### Cell lines.

Huh-7.5 (51), Huh-7.5.1 (52), and HEK293 cells were maintained in Dulbecco modified Eagle medium (DMEM) with 10% fetal bovine serum (FBS).

### Animals and cell lines.

Gt(ROSA)26Sor^tm1(Luc)Kaelin11^ (Rosa26-Fluc), B6; 129-Gt(ROSA)26Sor^tm1Joe^/J^27^ (Rosa26-GNZ), B6; 129S2-Scarb1^tm1Kri^/J^17^ (SCARB1^−/−^) mice were obtained from The Jackson Laboratory (Bar Harbor, ME) and Cd81^tm1Lvy^ (CD81^−/−^) mice were obtained from the European Mutant Mouse Archive (Munich, Germany). 129S6/SvEv-Stat1^tm1Rds^ (STAT1^−/−^) and 129S6/SvEvTac (wild-type) mice were obtained from Taconic (Germantown, NY). Human liver-uPA-SCID mice were generated as previously described (28, 53). Rosa26-Fluc mice contain the firefly luciferase (luc) gene inserted into the Gt.(ROSA)26Sor locus. Expression of the luciferase gene is blocked by a loxP-flanked STOP fragment placed between the luc sequence and the Gt.(ROSA)26Sor promoter. Cre recombinase-mediated excision of the transcriptional stop cassette results in luciferase expression in Cre-expressing tissues. Rosa26-GNZ knock-in mice have widespread expression of a nucleus-localized green fluorescent protein/beta-galactosidase fusion protein (GFP-NLS-GNZ) once an upstream loxP-flanked STOP sequence is removed. When Cre recombinase is introduced into cells, the resulting GNZ fusion protein expression allows for enhanced (single-cell level) visualization. All infections of mice with the indicated strains were performed intravenously.

Mice were bred and maintained at the Laboratory Animal Resources of Princeton University, Comparative Bioscience Center of the Rockefeller University, and at the Animal Resource Center of the University of Ghent according to guidelines established by the respective Institutional Animal Care and Use Committees.

### Hepatitis C virus.

The sequence of the genotype 2a/2a chimera J6/JFH1 has been deposited in a gene bank (accession number JF343782). Construction of Jc1 (54), and mCD81/Jc1 (24) (mtHCV) was described elsewhere. BiCre-Jc1/mCD81 was generated by introducing the L216F, V388G, and M405T point mutations into BiCre-Jc1 (16) via site-directed mutagenesis. Jc1(p7nsGluc2a) and Jc1/mCD81(p7nsGluc2a) were generated by ligating an EcoRI/BsaBI fragment containing core, E1, and parts of E2 into the EcoRI/BsaBI backbone of Jc1/Flag2(p7nsGluc2a) (29). To produce infectious virus, Huh-7.5.1 (52) or Huh-7.5 (55) cells were electroporated with *in vitro*-transcribed full-length HCV RNA (T7 RiboMAX express large-scale RNA production system; Promega, Madison, WI). Seventy-two hours postelectroporation, the medium was replaced with DMEM without FBS, and supernatants were harvested every 6 h starting from 72 h. Pooled supernatants were filtered through a 0.45-µm bottle top filter (Millipore, Billerica, MA) and concentrated using a stirred cell (Millipore). Viral titers (TCID_50_) were determined using Huh-7.5 cells as previously described (56).

### Adenoviruses.

Construction of adenoviral constructs encoding human or mouse CD81, SCARB1, CLDN1, or OCLN was described previously (16) using the AdEasy adenoviral vector system (Agilent Technologies, Santa Clara, CA) according to the manufacturer’s instructions. Briefly, cDNAs encoding HCV entry factors were PCR amplified and inserted into the pShuttle-CMV (CMV stands for cytomegalovirus) using KpnI/NotI sites. Recombinant pShuttle-CMV plasmids were linearized with PmeI and ligated to pAdEasy by homologous recombination followed by electroporation into BJ5183 cells (Agilent). Recombinant pShuttle-pAdEasy constructs were identified by PacI restriction analysis. All plasmid constructs were verified by DNA sequencing.

### RT-PCR quantification of HCV RNA.

Total RNA was isolated from the indicated mouse tissues using the RNeasy kit (Qiagen, Valencia, CA) and from serum using the QIAamp viral RNA minikit. HCV genome copy number was quantified by one-step reverse transcription-PCR (RT-PCR) using MultiCode ISOlution (catalog no. 3691; Luminex, Madison, WI), reverse transcriptase (catalog no. 3693; Luminex, Madison, WI), Titanium Taq (catalog no. S1792; Clontech, Mountain View, CA), and a Step One Plus quantitative PCR (qPCR) machine (Life Technologies, Carlsbad, CA), according to manufacturers’ instructions. Data were analyzed using the MultiCode Analysis Software v1.6.5 (Luminex, Madison, WI). The following primers were used for the detection of HCV RNA: GCTCACGGACCTTTCA (sense) and GGCTCCATCTTAGCCC (antisense).

Quantification of HCV RNA in the chimeric Alb-uPA mice was performed with the Roche COBAS AmpliPrep instrument and the COBAS TaqMan 48 HCV test.

### Antibodies.

Blocking antibodies against CD81 (JS81) and IgG1 control antibodies were obtained from BD Biosciences (Franklin Lakes, NJ). Antibodies against NS5A (56) and E2 (clone AR4A) (33) and the human IgG1 isotype control (B12) (57) have been described previously. As a secondary antibody for the detection of anti-NS5A, Alexa Fluor 647-labeled goat anti-mouse IgG (Life Technologies, Carlsbad, CA) was used.

### Flow cytometry.

The frequency of infected hepatocytes and the frequency of infected Huh-7.5 cells after inoculation with sera from infected mice were confirmed by flow cytometry using an LSRII flow cytometer (BD Biosciences). For the determination of infection frequency, hepatocytes were isolated from Rosa26-GNZ mice, fixed in 4% paraformaldehyde, permeabilized in phosphate-buffered saline (PBS) plus 0.01% Triton X-100, stained with anti-NS5A, and counterstained with DRAQ5. Data were analyzed using FlowJo software (Treestar Software, Ashland, OR).

### Isolation and infection of murine hepatocytes.

C57BL/6 mice were anesthetized by intraperitoneal injection of a mixture of 100 mg of ketamine/kg of body weight and 10 mg/kg xylazine. Livers were perfused through the portal vein with a chelating solution (0.01 M HEPES [pH 7.3] and 0.5 mM EGTA [pH 8.0] in Ca^2+^/Mg^2+^-free Earle’s balanced salt solution [EBSS]) at a flow rate of 2 ml/min until the liver bleached, followed by 40 ml of collagenase solution (0.01 M HEPES [pH 7.3] and 1 mg/ml collagenase type II in EBSS with Ca^2+^, Mg^2+^, and phenol red). The digested liver was cut into pieces, transferred into a washing solution (0.01 M HEPES [pH 7.3] and 10% FBS in DMEM), passed through a 100-µm cell strainer, washed, and passed through a 100-µm cell strainer. The resulting cell suspension was passed through a 70-µm cell strainer. The cell suspension was washed three more times with spinning steps at 140 × *g* for 5 min to remove unwanted cellular contaminants. The cells were resuspended in HBM basal medium after adding HCM Single Quots (Lonza, Basel, Switzerland), counted, and seeded on collagen-coated 24-well cell culture plates (Corning, Corning, NY) at a density of 1.25 × 10^5^ cells/cm^2^. The medium was changed every other day. Confluent monolayers of primary hepatocytes were infected with different doses of Jc1(p7nsGluc2A) or Jc1/mCD81p7nsGluc2A for 12 to 18 h and washed 3 or 4 times with medium, and supernatants were collected at 48 h following infection. *Gaussia* luciferase activity was quantified in the cell culture supernatants with a luciferase assay system (Promega, Madison, WI) according to the manufacturer’s instructions using a Tristar 2 multimode reader LB942 (Berthold Technologies, Bad Wildbad, Germany).

### Bioluminescence im aging.

Unless otherwise specified, mice were injected with 10^11^ adenoviral particles 24 h prior to intravenous injection with 2 × 10^7^ TCID_50_ HCV-Cre. At 72 h postinfection, mice were anesthetized using ketamine/xylazine and injected intraperitoneally with 1.5 mg Luciferin (Caliper Life Sciences, Hopkinton, MA). Bioluminescence was measured using an IVIS Lumina II platform (Caliper Life Sciences).

### HCV genome sequencing.

Serum samples from mice inoculated with Jc1/mCD81 were subjected to RNA purification (high pure viral RNA kit; Roche, Basel, Switzerland) and cDNA amplification (Transcriptor high-fidelity cDNA synthesis kit; Roche, Basel, Switzerland) with an antisense primer situated on the NS2 gene. cDNA content was determined by qRT-PCR. For amplicon library preparation, six contigs which covered the signal peptide genes of E1, E2, and p7 (amino acids 131 to 813, corresponding to the polyprotein sequence of the reference strain Jc1) were generated with the Expand Long Range dNTPack kit (Roche, Basel, Switzerland) by nested PCR utilizing 12 primers tagged at the 5′ end. Amplicons were prepared individually and quantified by using the Quant-iT PicoGreen dsDNA (double-stranded DNA) assay kit (Carlsbad, CA). Emulsion-PCR and bidirectional sequencing reaction was performed using the 454 platform (Roche, Basel, Switzerland) as described in the GS FLX emPCR Methods Manual (Roche, Basel, Switzerland). Sequencing data were analyzed using CLC genomics workbench v4.9 (Aarhus, Denmark). Contig assembly and viral population variant calling were performed as described previously (58). The average read depth was 11,000, ranging from 3,000 to 21,000 reads.

### Statistical analysis.

Statistical analysis was performed with GraphPad Prism software (La Jolla, CA). Statistics were calculated using *t* test or Kruskal-Wallis one-way analysis of variance (ANOVA). *P* values less than 0.05 were considered statistically significant.
